# Planning of the nursing team in the management of multiple births: from medically assisted reproduction center to neonatal intensive care unit

**DOI:** 10.1186/1824-7288-40-S2-A46

**Published:** 2014-10-09

**Authors:** Martina Cecconi, Laura Ligi, Assunta Fabi, Luisa Pieragostini

**Affiliations:** 1 Neonatal Intensive Care Unit, San Filippo Neri Hospital, Rome, Italy

## Background

In the last twenty years the number of multiple births in Italy growed up to around 25% due to the increase of medically assisted reproduction. Multiple birth usually occurs between the 34^th^ and the 37^th^ week of gestational age, that coincides with the "Late-Preterm" newborns. For theirs peculiarities, late-preterm newborns require more assistance and care [1-3].

## Aim

In this study we tried to leave procedures based largely on tradition and “commonsense”, burdened by wide variability of pediatric team. We tried to organize a shared and standardized procedural planning, according to the evidence-based practices criteria and the operative context, improving the safety level of the newborns and team.

The collaboration between different professionals, from obstetric to neonatal care area, is necessary in order to ensure an interdisciplinary and highly specialized standard of care.

## Materials and methods

After approbation of the Hospital Committee we planned a scheduled care process that provides:

a) Early stage of training: acquisition of care pathways encoded for every professional involved, including participation in training specialized courses and simulation cases;

b) The establishment of a multidisciplinary study group that defines the indicators and the standard of result to be achieved, including the emotional and psychological aspect of the new mother and the identification of the role of the various members of the team;

c) Strict planning and management of human resources and equipment available, listed in detail and sequence of use, updated with the latest national legislative directives;

d) Estimates of possible variables and complications; identification of properly safety systems and alternative recommendations to be observed, not covered by the standard procedure;

e) Insertion of a self-assessment system in order to check the feasibility and the adherence to the protocol.

## Results

We performed a procedural planning that ensures the standardization and uniformity of the nursing of the multiple late preterm newborn, managing the collaboration between several professionals in order to minimize adverse and unexpected events.

## Conclusions

The quality of the health care level provided depends on the cooperation of the professionals. The management of the team work allows to ensure an optimal and personalized care level from the medically assisted reproduction center to the neonatal intensive care unit, following the updated scientific evidence and the humanization of birth route.
